# Recognition memory in developmental prosopagnosia: electrophysiological evidence for abnormal routes to face recognition

**DOI:** 10.3389/fnhum.2014.00622

**Published:** 2014-08-14

**Authors:** Edwin J. Burns, Jeremy J. Tree, Christoph T. Weidemann

**Affiliations:** Department of Psychology, Swansea UniversitySwansea, Wales, UK

**Keywords:** prosopagnosia, face recognition, recognition memory, familiarity, recollection, electroencephalogram (EEG)

## Abstract

Dual process models of recognition memory propose two distinct routes for recognizing a face: recollection and familiarity. Recollection is characterized by the remembering of some contextual detail from a previous encounter with a face whereas familiarity is the feeling of finding a face familiar without any contextual details. The Remember/Know (R/K) paradigm is thought to index the relative contributions of recollection and familiarity to recognition performance. Despite researchers measuring face recognition deficits in developmental prosopagnosia (DP) through a variety of methods, none have considered the distinct contributions of recollection and familiarity to recognition performance. The present study examined recognition memory for faces in eight individuals with DP and a group of controls using an R/K paradigm while recording electroencephalogram (EEG) data at the scalp. Those with DP were found to produce fewer correct “remember” responses and more false alarms than controls. EEG results showed that posterior “remember” old/new effects were delayed and restricted to the right posterior (RP) area in those with DP in comparison to the controls. A posterior “know” old/new effect commonly associated with familiarity for faces was only present in the controls whereas individuals with DP exhibited a frontal “know” old/new effect commonly associated with words, objects and pictures. These results suggest that individuals with DP do not utilize normal face-specific routes when making face recognition judgments but instead process faces using a pathway more commonly associated with objects.

## Introduction

Prosopagnosia is a selective face perception disorder characterized by an impairment for recognizing faces combined with intact low level visual processing (Bodamer, 1947). It had been thought until recently that prosopagnosia was a rare disorder, with the vast number of identified cases acquiring problems with face recognition following some form of brain injury (Farah, 1990). However, cases with no evidence of neurological injury have been identified in recent years (e.g., de Haan, 1999; Duchaine, 2000; Duchaine et al., 2003). These latter cases have become known as Congenital or Developmental Prosopagnosia (DP). It has been suggested that as many as 1 in 40 of the population meets the criteria for DP (Kennerknecht et al., 2006), with some cases appearing to run in families (Duchaine et al., 2007; Grueter et al., 2007). While individuals with DP exhibit difficulties in recognizing faces, many, but not all, have been shown to possess normal attractiveness processing (Carbon et al., 2010), as well as intact recognition abilities for eye gaze (Duchaine et al., 2009), face emotion (Duchaine et al., 2003; Humphreys et al., 2007), face motion information (Steede et al., 2007; Longmore and Tree, 2013) and greebles (artificial objects designed to be processed holistically like a face; Duchaine et al., 2004).

Face recognition deficits associated with prosopagnosia have been studied using a wide variety of methods: forced choice tasks (e.g., Duchaine and Nakayama, 2006; Rivolta et al., 2012), familiarity judgments (e.g., Kress and Daum, 2003; Grueter et al., 2007) or recall tests for semantic information related to faces such as a name or profession (e.g., Grueter et al., 2007). Dual process models of recognition memory (e.g., Atkinson and Juola, 1973, 1974; Mandler, 1980; Jacoby, 1991; Yonelinas, 1994) propose that there are two distinct routes with which one can recognize a previously seen face: familiarity and recollection. Most of us can relate to the experience of meeting someone and finding their face familiar but, rather frustratingly, being unable to remember any details from when or where one might have met them; this is an example of familiarity based recognition. Recollection on the other hand is characterized by remembering some form of contextual detail, such as specific previous encounters. Traditional dual process models propose that familiarity can vary in strength whereas recollection is usually assumed to be an all-or-nothing, high strength memory (Yonelinas, 2002; for an alternative perspective on the nature of recollection, see Donaldson, 1996; Wixted, 2007; Wixted and Mickes, 2010).

A raft of behavioral, neuropsychological, electrophysiological and neuroimaging studies have provided evidence in support of this dissociation between familiarity and recollection (for reviews, see Yonelinas, 2002; Aggleton and Brown, 2006; Diana et al., 2007). One behavioral method for dissociating familiarity and recollection is the Remember/Know (R/K) procedure (Tulving, 1985). Participants are asked to study a series of items and are then tested on the studied target items along with previously unknown lures. Participants are required to make judgments of “Remember”, that is if they could recollect some detail of the item from study, “Know”, where they knew they had seen the item in the previous list but could not recollect any details of its presentation or “New”, an item that was not on the previous list. It is thought that “remember” responses reflect the recollection process whereas “know” responses measure the contribution of familiarity (Yonelinas, 2002). This suggests that remember responses are associated with high confidence due to the high strength of memory that recollecting details surrounding an item’s previous occurrence brings (Eichenbaum et al., 2007). Know responses, however, engender a more pliable level of confidence due the fact familiarity can vary in memory strength (Eichenbaum et al., 2007) The R/K procedure has been successful in dissociating recollection and familiarity effects in electrophysiological (Düzel et al., 1997) and neuroimaging studies (Henson et al., 1999). The present study is the first to use the R/K paradigm to study the recognition of previously unknown faces in individuals with DP.

Traditionally, event related potential (ERP) studies of pictures (e.g., Tsivilis et al., 2001), objects (e.g., Duarte et al., 2004; Groh-Bordin et al., 2006) and words (e.g., Curran, 2000; Maratos et al., 2000) have found familiarity to be associated with early enhanced positivity over frontal regions between 300–500 ms after test stimulus onset, whereas later positivity over parietal sites between 500–700 ms indicates recollection. However, recent ERP studies examining recognition memory for previously unknown faces have suggested that familiarity and recollection might differ temporally and neurally to that of words and objects (Yovel and Paller, 2004; MacKenzie and Donaldson, 2007; Herzmann et al., 2011). These results contribute to the ample evidence suggesting that faces are special stimuli processed differently from other objects (for a review, see McKone and Robbins, 2011). By using an adapted R/K procedure, Yovel and Paller (2004) found that familiarity for faces was associated with a parietal old/new effect between 300–700 ms, whereas recollection for faces was associated with similar positivity over the posterior of the scalp, but also some anterior regions during the same time period. Recollection and familiarity were also found to be maximal between 500–700 ms after stimulus onset. A study by MacKenzie and Donaldson (2007) also found spatially and temporally similar familiarity and recollection ERP effects for faces. In contrast to these studies, Curran and Hancock (2007) found face related ERP effects similar to that of words, pictures and objects. These results might be due to their participants recognizing face images on the basis of extraneous information in the images rather than the facial features. In a follow-up study, Herzmann et al. (2011) showed ERP effects for faces in line with earlier work cited above when extraneous cues were excluded from face images. These results suggest that the removal of any such extraneous cues from face images is important for the study of face processing and consistent with previous work showing that general object processing can be dissociated from that of faces (McNeil and Warrington, 1993; Farah et al., 1995; Moscovitch et al., 1997).

The present study examines recognition memory for faces in those with normal face recognition abilities and individuals with DP in order to determine the relative contributions of recollection and familiarity to performance in these two groups. Moreover, the use of electroencephalogram (EEG) measures enables us to determine the degree to which differences in performance across these two groups reflect qualitative (rather than just quantitative) differences in face processing.

## Methods

### Participants

Eight individuals with DP and 20 control participants took part in this study. Four of the individuals with DP and 11 of the control participants were female. The ages of the individuals with DP ranged from 20–38 years (*M* = 25.6 years) and that of the control participants ranged from 18–40 years (*M* = 24.5 years). All participants had normal or corrected to normal vision. One of the individuals with DP and 2 of the control participants were left-handed. Data from 1 control participant was rejected from all analyses due to behavioral performance appearing to be at chance levels. Nine controls failed to correctly respond “know” on enough trials to create reliable ERP waveforms for these responses and were excluded from the ERP analyses described below (we confirmed that their ERPs for correct “remember” responses matched those for the remaining 10 controls and their choice responses are included in Tables 1–4). The ERPs for the control group are based on 5 male and 5 female participants between the ages of 19 and 40 years (*M* = 27.9) one of which was left handed. Ethical approval for this study was granted by the departmental Ethics Committee at Swansea University.

**Table 1:** **Neuropsychological testing results of the 8 DP cases: Famous Faces Test (FFT), Cambridge Face Memory Test (CFMT), Cambridge Face Perception Test upright and inverted (CFPTupr and CFPTinv)**.

**Participants**	**Age**	**Sex**	**FFT**	**CFMT**	**CFPTupr**	**CFPTinv**
			(%)	*z*	*z*	*z*
DP1	32	M	66	−2.77	−1.25	1.09
DP2	21	M	60	−2.27	−1.91	0.15
DP3	20	M	63	−2.84	−3.06	−1.47
DP4	38	M	31	−3.24	−3.88	−0.95
DP5	20	F	29	−2.92	−2.24	−0.5
DP6	21	F	26	−3.19	−2.24	−0.8
DP7	21	F	34	−2.15	−0.93	1.8
DP8	32	F	46	−2.99	−3.55	−2.14

**Table 2:** **Mean accuracy and proportion of correct and incorrect responses (with standard errors)**.

	**Controls (%)**	**DP Cases (%)**
Hits	93 (1.23)	79 (3.42)
False Alarms	22 (2.72)	42 (3.32)
Correct:		
Remember	74 (4.46)	52 (5.61)
Know	26 (4.46)	48 (5.61)
Incorrect:		
Remember	25 (5.89)	13 (2.74)
Know	75 (5.89)	87 (2.74)

**Table 3:** **Discriminability (with standard errors)**.

	**Controls**	**DP Cases**
Discriminability	2.38 (0.16)	1.09 (0.13)
Discriminability:
Remember	2.36 (0.24)	1.45 (0.12)
Know	0.24 (0.17)	0.02 (0.08)

**Table 4:** **Mean response times (RTs) of correct and incorrect responses in ms (standard errors)**.

	**Controls**	**DP Cases**
Correct:
Remember	839 (108)	657 (117)
Know	1420 (180)	1041 (130)
Incorrect:
Remember	1473 (268)	802 (87)
Know	1761 (212)	1032 (155)

In line with previous researchers (Duchaine et al., 2007; Bate et al., 2008), we used a battery of neuropsychological tests (described in detail below) to diagnose DP. Unless noted otherwise, we took the appropriate norms from the respective research publications. Table 1 displays the DP cases that participated in this experiment and their neuropsychological tests of face processing impairment. The Famous Faces Test (FFT; Duchaine and Nakayama, 2005) consists of 60 celebrity faces which the participant is required to name or identify in some way. We collected FFT data from 164 participants (101 female) using a shortened FFT (35 faces) in a separate study from the present one to ascertain normative means and SDs for the general population in the local geographical area (*M* = 94.6%, *SD* = 6.23). As can be seen from Table 1, all of the DP cases were severely impaired at recognizing famous faces. The Cambridge Face Memory Test (CFMT; Duchaine and Nakayama, 2006) requires the participant to memorize six target faces presented in a number of different views; these faces must then be identified when displayed individually with two distractor faces. We only recruited DP cases that showed an impairment of two SDs or more below the mean in both the CFMT and FFT. During the Cambridge Face Perception Test (CFPT; Duchaine et al., 2007), participants are shown a target face presented in three-quarter view along with six faces presented in frontal view; these six faces have been morphed to appear similar in varying percentages to the target face. Participants are required to arrange the faces in order of similarity to the target face. The test displays faces either upright or inverted. As can be seen from Table 1, five of the DP participants were impaired on the CFPT with a sixth case approaching 2 SDs below the mean; it should be noted that a diagnosis of prosopagnosia is not reliant upon impairment on this task. We also screened control participants for prosopagnosia by administering the CFMT and confirmed that all *z*-scores were within the normal range (−1.5–1.4, *M* = −0.36).

### Stimuli

**Figure 1:**
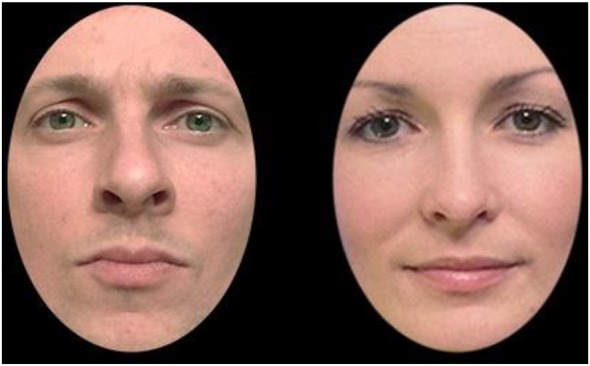
**Mock-up examples of the male and female face stimuli used**.

Experimental stimuli consisted of 324 photographic bitmap images of faces, half of which were male. Figure 1 shows mock-up examples of two such stimuli. All faces were unknown to the participants. The faces were presented in the center of a black background on a 14″ color monitor. The stimuli subtended horizontal and vertical visual angles of approximately 3.9° and 5.4° respectively. In addition, each face was masked to remove the original background, hair, and ears, i.e., cues that could lead to recognition not based upon the face itself. Luminance of each face was homogenized for the same purpose.

### Procedure

Following application of electrodes (described below), participants were seated on a comfortable chair in a dimly lit booth. The participants faced a computer screen at a distance of approximately 90 cm, with the response buttons placed comfortably within reach to record responses. Participants were fully instructed prior to a practice session consisting of a study and test phase. Before the beginning of any study or test phase, the instructions for each task were repeated to remind participants as to what was required. Between phases, participants were also reminded to remain as still as possible and to fixate centrally throughout stimulus presentation.

The experiment was comprised of 27 blocks of study and test lists. At study participants were asked to remember the faces as best they could and were told that their memory for the faces would be tested in a subsequent test phase. In each study phase participants viewed four repetitions of six face images (half of which were male) for a total of 24 trials. Presentation of the face images was random subject to the constraint that all six faces had to be presented before the next round of repetitions and that no faces repeated across blocks. Each trial consisted of a white fixation cross presented for either 450 or 550 ms, followed by the presentation of a face image for 2500 ms. A 500 ms blank screen then followed prior to the presentation of the next trial.

All faces displayed during the previous study phase, and the six new faces were presented in a random order at test (subject to the constraint that no faces repeated across blocks). Participants were asked to decide whether each face had been presented in the previous study phase, or not, by pressing “remember” if they could remember specific details from the study phase, “know” if they thought the face was encountered in the previous study phase but without remembering any details, or “new” with the first three fingers of their dominant hand (the mapping between buttons and responses was counterbalanced across all participants). Each trial consisted of a white fixation cross presented for either 450 or 550 ms, followed by the presentation of a face image for 2000 ms. Following the face, a white fixation cross would appear again for 150 ms and then a screen prompting participants to respond “remember”, “know” or “new” would appear; this screen would remain on screen until a response was made. Participants could not respond until this response prompt screen had appeared. After a response was made, another fixation screen would appear for 150 ms followed by another screen prompting participants to rate on a scale of 1–6 how confident they were of their previous response.

### EEG recording

We recorded electrophysiological data throughout the experiment. The recording at scalp was taken from 128 Ag-AgCl “active” electrodes set in an elastic Biosemi (Amsterdam, the Netherlands) cap. Each electrode was set within the cap in equidistant concentric circles from the 10 to 20 position Cz (Jasper, 1958). The horizontal electro-oculogram (EOG) was recorded from electrodes placed on the outer canthi of each eye. The vertical EOG was recorded from an electrode placed below the left eye. The EEG was recorded referenced to a common mode sense (CMS) electrode, and then re-referenced offline to a common average reference through the use of Brain Electrical Source Analysis (BESA) software (MEGIS software GmbH, Graefelfing, Germany). All electrode channels were band pass filtered from 0.01 to 40 Hz. The analogue signal was digitally sampled at a rate of 512 Hz. ERPs were time locked to the presentation of stimuli, with an epoch that began 200 ms prior to stimulus onset and lasted for 1000 ms post-stimulus. Epochs found to contain EOG artifacts exceeding ±100 μV were rejected from analysis, as were trials where drift from baseline (difference between first and last data point) was greater than 50 μV. We retained data only from those participants with at least 20 remaining trials in each of the experimental conditions of interest. Blink artifacts were corrected using the algorithm implemented in BESA (Berg and Scherg, 1994).

## Results

### Behavioral results

Table 2 displays the percentage of hits, that is the correct identification of a studied face as studied, from the control and DP participants. Between samples* t*-tests comparing the two groups revealed significant differences for the hits [*t*_(25)_ = 4.52, *SE* = 2.89, *p* = 0.009], suggesting that the control participants were better at identifying studied faces as having been previously seen when compared to the individuals with DP. The mean proportion of response types for hits for the controls and those with DP are also shown in Table 2. A mixed within-between subject ANOVA of Group (DP, control) × Response (“remember”, “know”) revealed a significant Group × Response interaction [*F*_(1, 25)_ = 7.84, *MSE* = 5363.29, *p* = 0.01] and a significant effect of Response [*F*_(1, 25)_ = 10.74, *MSE* = 7346.11, *p* = 0.003]. Paired samples *t*-tests revealed that the control participants made significantly more “remember” than “know” responses when correctly identifying an old face as previously seen [*t*_(18)_ = 5.315, *SE* = 8.91, *p* < 0.001], and no significant differences in response proportions for individuals with DP [*t*_(7)_ = 0.332, *SE* = 11.22, *p* = 0.75]. Between samples *t*-tests revealed significant differences between the individuals with DP and control participants in their proportion of “remember” responses [*t*_(25)_ = 2.8, *SE* = 7.79, *p* = 0.01]. These results show that when control participants correctly identified previously studied faces, they did so more frequently using “remember” responses than individuals with DP.

Table 2 also displays the percentage of false alarms, that is the incorrect identification of a previously unknown lure face as studied, from the control and DP participants. Between samples *t*-tests comparing the two groups revealed significant differences for the false alarms [*t*_(25)_ = −4.21, *SE* = 4.73, *p* < 0.001], suggesting that the DP participants were more likely to identify an unstudied face as studied in comparison to the controls. Also displayed in Table 2 is the mean proportion of incorrect identification of test faces as studied (false alarms). A mixed within-between subject ANOVA of Group (DP, control) × Response (“remember”, “know”) revealed a significant effect of response [*F*_(1,25)_ = 44.26, *MSE* = 43514.79,* p* < 0.001]. Paired samples *t*-tests revealed that both groups were more likely to incorrectly identify a previously unknown face as being studied using a “know” response rather than a “remember” response [*t*_(18)_ = 4.247, *SE* = 11.78, *p* < 0.001, and *t*_(7)_ = 13.568, *SE* = 5.48, *p* < 0.001], for control participants and individuals with DP respectively.

Table 3 displays the mean discriminability (hits—false alarms; Donaldson, 1996). A discriminability score of 0 corresponds to no discrimination between studied and new items. A between samples *t*-test revealed significant differences in discriminability between the DP and control participants [*t*_(25)_ = 4.98, *SE* = 0.25, *p* < 0.001]. This suggests that individuals with DP found it harder than controls to discriminate between old and new faces. Between samples *t*-tests also revealed that for “remember” responses, control participants were more effective than those with DP at discriminating old and new faces [*t*_(25)_ = 2.78, *SE* = 0.35, *p* = 0.01], whereas we found no difference in discriminability for “know” responses [*t*_(25)_ = 0.82, *SE* = 0.27, *p* = 0.42]. One sample *t*-tests revealed that “remember” responses significantly discriminated old and new faces [*t*_(18)_ = 11.11, *SE* = 2.42, *p* < 0.001, and *t*_(7)_ = 12.48, *SE* = 1.45, *p* < 0.001], for the control and DP participants respectively. Neither group, however, reliably discriminated old and new faces when responding “know” [*t*_(18)_ = 1.43, *SE* = 0.24, *p* = 0.169, and *t*_(7)_ = 0.392, *SE* = 0.35, *p* = 0.78], for the control and DP participants respectively.

The response times for correct “remember” and “know” responses across the two groups are displayed in Table 4. A mixed within-between subject ANOVA of Group (DP, control) × Response (“remember”, “know”) revealed a significant Group × Response interaction [*F*_(1,25)_ = 12.86, *MSE* = 2622262.51, *p* = 0.001]. Within groups *t*-tests revealed that control participants and individuals with DP responded significantly faster with “remember” than “know” for previously studied faces [*t*_(18)_ = −3.46, *SE* = 169.75, *p* = 0.003, and *t*_(7)_ = −4.919, *SE* = 78.04, *p* = 0.002, respectively]. There were no significant response time differences between the two groups for correct “remember” [*t*_(25)_ = 0.99, *SE* = 183.24, *p* = 0.23], and correct “know” [*t*_(25)_ = 1.29, *SE* = 292.85, *p* = 0.21], responses.

Table 4 also displays the incorrect “remember” and “know” responses across the two groups. A mixed within-between subject ANOVA of Group (DP, control) × Response (“remember”, “know”) revealed no significant effects of Response [*F*_(1,25)_ = 2.53, *MSE* = 729116, *p* = 0.126], or Response × Group [*F*_(1,25)_ = 0.032, *MSE* = 9306, *p* = 0.86]. Pairwise comparisons revealed no significant differences between response times for incorrect “remember” responses across groups [*t*_(23)_ = 1.68, *SE* = 0.400, *p* = 0.11], but individuals with DP made incorrect “know” responses significantly faster than the control participants [*t*_(25)_ = 2.148, *SE* = 322, *p* = 0.04]. Pairwise comparisons revealed that the correct “remember” responses were faster than incorrect “remember” responses in the control group [*t*_(16)_ = 3.16, *SE* = 208, *p* = 0.006], but not the DP group [*t*_(7)_ = 1.11, *SE* = 131, *p* = 0.3]. There were no significant differences between response times for correct vs. incorrect “know” responses in either the controls [*t*_(18)_ = 1.38, *SE* = 221, *p* = 0.18], or DP group [*t*_(7)_ = 0.128, *SE* = 68, *p* = 0.9].

Overall, the pattern of performance for the two groups in this task suggest that (a) recognition memory for faces in individuals with DP was clearly impaired relative to the control participants; (b) control participants showed the typical pattern of a greater proportion of “remember” than “know” responses (consistent with other work: Yovel and Paller, 2004; MacKenzie and Donaldson, 2007, 2009); whilst (c) individuals with DP showed no preference for “remember” responses.

### Electrophysiological results

#### ERP effects commonly associated with recognition memory for faces

**Figure 2:**
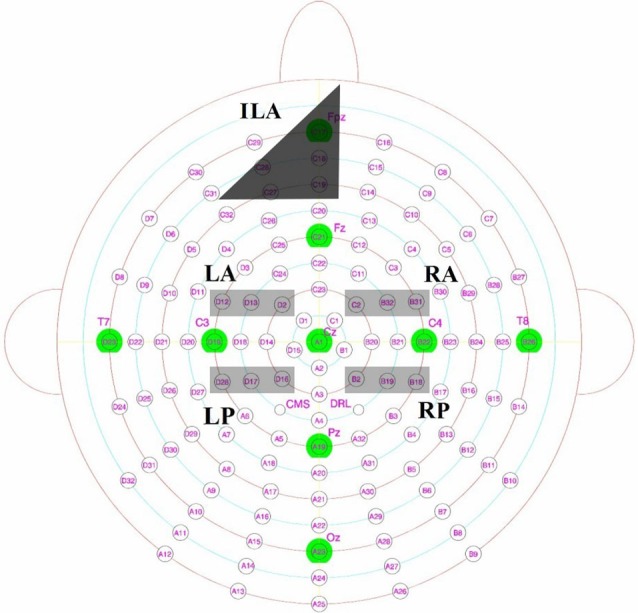
**Biosemi electrodes with key Jasper 10–20 locations overlaid, with left posterior (LP), left anterior (LA), right anterior (RA) and right posterior (RP) sites highlighted in light gray**. Inferior mid left anterior (ILA) as described from the scalp maps is shown in dark gray.

For analyses, we divided the central scalp area into four a-priori regions of interest at time intervals of 300–500 ms and 500–700 ms (c.f., Yovel and Paller, 2004; MacKenzie and Donaldson, 2007) as recollection and familiarity for faces were previously found to occur across both these time windows. The main regions of focus will be across the left and right hemispheres from anterior (left hemisphere: D2, D12, D13; right hemisphere: C2, B31, B32) and posterior sites (left hemisphere: D16, D17, D28; right hemisphere: B2, B18, B19). These electrodes were chosen as they would capture the enhanced positivity exhibited for familiarity and recollection of faces as identified by previous research (Yovel and Paller, 2004; MacKenzie and Donaldson, 2007). These sites would also allow us to examine possible topographical differences between where these effects occur in those with DP and intact face recognition skills. Figure 2 displays the locations of these electrodes in the Biosemi cap system.

#### ANOVAs from the four scalp locations

We performed mixed within-between subject ANOVAs with factors of Correct Response (remember, know, correct rejections), Location (anterior, posterior), Hemisphere (left, right) and Group (control, DP) on the data from the 300–500 ms and 500–700 ms time windows. In the 300–500 ms time window we found a main effect of Location [*F*_(1,16)_ = , *MSE* = 31.71, *p* = 0.001], and a significant interaction for Location × Hemisphere [*F*_(1,16)_ = 12.84, *MSE* = 7.51, *p* = 0.002] and Response × Hemisphere × Group interaction [*F*_(2,32)_ = 2.9, *MSE* = 0.73, *p* = 0.069]. In the latter time window (500–700 ms), we found a main effect of Location [*F*_(1,16)_ = 20.11, *MSE* = 43.14, *p* < 0.001] and Response [*F*_(2,32)_ = 6.77, *MSE* = 7.58, *p* = 0.004], and a significant interaction for Location × Response [*F*_(2,32)_ = 5.29, *MSE* = 0.97, *p* = 0.01], Location × Hemisphere [*F*_(2,16)_ = 6.6, *MSE* = 5.71, *p* = 0.021], Response × Hemisphere (Mauchly’s Test of Sphericity indicated that the assumption of sphericity had been violated, *χ*^2^(2) = 7.96, *p* = 0.019, therefore degrees of freedom were corrected using Greenhouse-Geisser estimates of sphericity (*ɛ* = 0.71)) [*F*_(1.42,22.67)_ = 3.56, *MSE* = 1.67, *p* = 0.059] Response × Hemisphere × Group interaction [*F*_(2,32)_ = 3.48, *MSE* = 1.16, *p* = 0.043]. The following sections contain pairwise comparisons that reveal the causes of these effects.

#### ERP effects commonly associated with familiarity for faces

**Figure 3:**
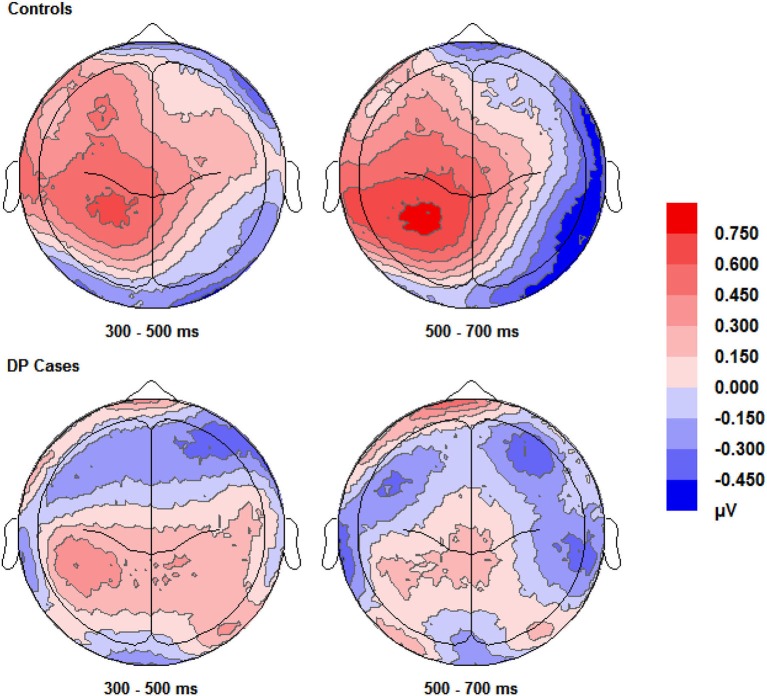
**Scalp maps (shown as if viewing from above the head) for the voltage corresponding to correct “know” responses minus that for correct rejections**. Data for both groups and time intervals are shown across the four panels.

Figure 3 shows enhanced positivity over the posterior and anterior scalp regions, particularly over the left hemisphere, for control participants when they correctly responded “know” compared to correct “new” responses. The DP cases display some faint positivity over the central and posterior of the scalp across 300–700 ms, however this positivity appears hugely diminished and covers less of the scalp anterior in comparison to the controls.

Examining the ERP waveforms in Figure 5, controls appear to show enhanced positivity for correct “know” responses from around 200–1000 ms when compared to correct rejections, but only over the left hemisphere. While DP cases display some positivity for correct “know” responses from around 300–400 ms in all scalp areas, this positivity only lasts until 600–700 ms, and is of smaller magnitude when compared to that of the controls.

**Figure 4:**
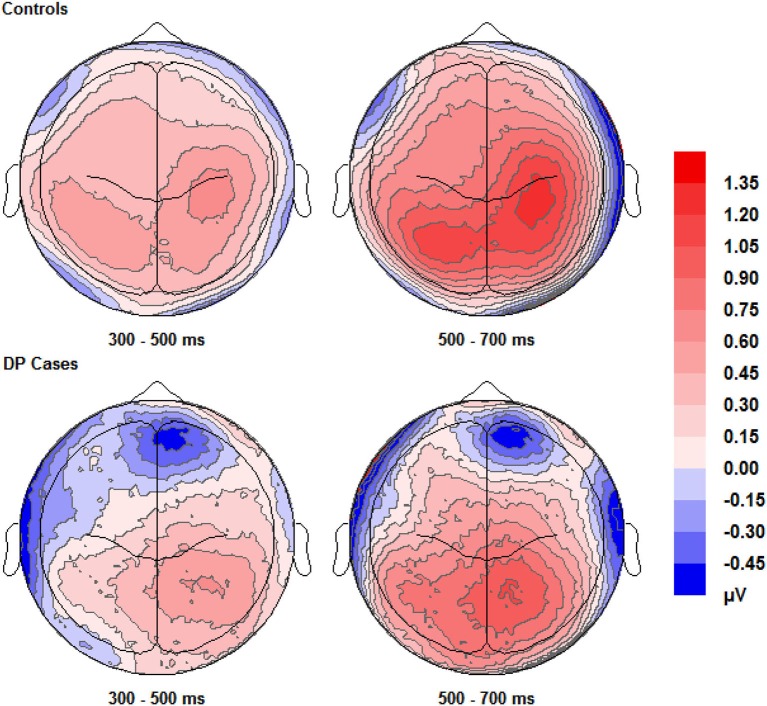
**Scalp maps (shown as if viewing from above the head) for the voltage corresponding to correct “remember” responses minus that for correct rejections**. Data for both groups and time intervals are shown across the four panels.

**Figure 5:**
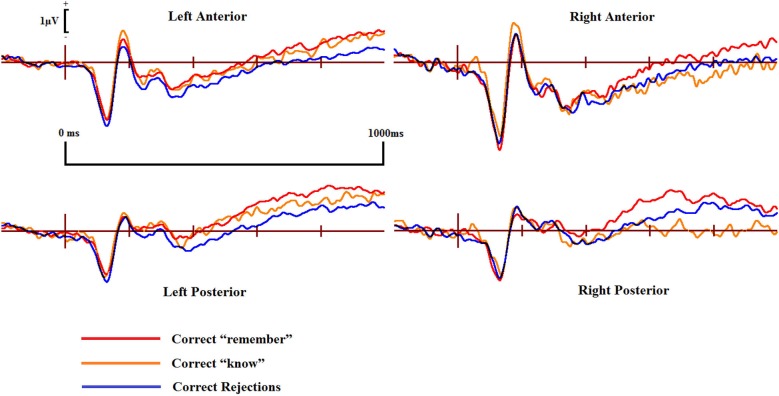
**ERPs of correct “remember” and “know” responses compared to correct rejections from the four scalp locations for the controls**.

Pairwise comparisons at each scalp location from the 300–500 ms time window revealed that the control group’s correct “know” [*t*_(9)_ = 2.39, *SE* = 0.207, *p* = 0.038] responses were more positive than correct rejections at the left posterior (LP) region. The DP group exhibited no such positivity over any scalp location in this time period.

In the 500–700 ms time window, pairwise comparisons revealed that in control participants, ERPs over the LP area for correct “know” responses were more positive than those for correct rejections [*t*_(9)_ = 2.656, *SE* = 0.254, *p* = 0.026]. Again, as in the earlier time window, the DP group exhibited no apparent “know” old/new effects in any of the four scalp locations. In addition, we found a significant difference between the groups when the mean amplitude of ERPs for correct rejection responses was subtracted from that for correct “know” responses at the LP site [*t*_(16)_ = 2.168, *SE* = 0.382, *p* = 0.046], suggesting a greater old/new effect for correct “know” responses in the control participants in the later time window.

ERPs for correct “know” responses are more positive relative to that of the correct rejections over the LP region in the controls in both time windows. This suggests that the controls are experiencing a similar face-specific familiarity old/new effect as found by previous research (Yovel and Paller, 2004; MacKenzie and Donaldson, 2007). In contrast, our results suggest that for individuals with DP, ERPs in the four central scalp regions do not distinguish between correct “know” responses and correct rejections; there is no expected face-specific familiarity signal present in the DP group.

#### ERP effects commonly associated with recollection for faces

Figure 4 shows that EEG voltage for correct “remember” responses is more positive than that for correct rejections across the whole scalp for both of the two participant groups. This difference appears maximal over the right hemisphere’s central area and is more pronounced in control participants than in individuals with DP.

**Figure 6:**
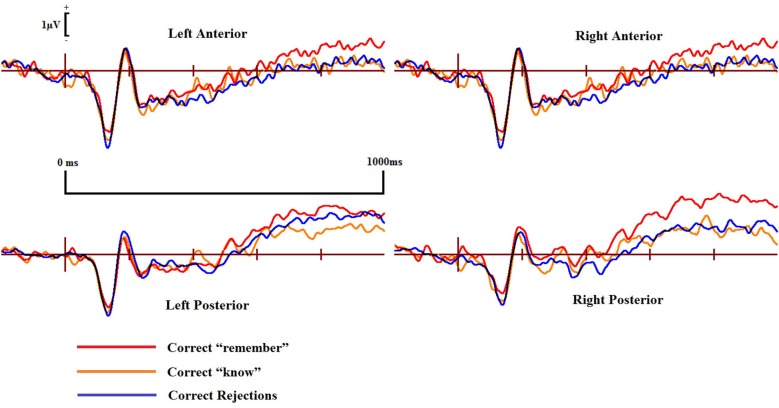
**ERPs of correct “remember” and “know” responses compared to correct rejections from the four scalp locations for the DP cases**.

ERPs for the four main regions of interest are shown in Figures 5 and 6 for the control and the DP participants respectively. Figure 5 suggests that control participants exhibit enhanced positivity for correct “remember” responses when compared to correct rejections from around 200–300 ms until the end of the epoch at 1000 ms over all four scalp regions. Individuals with DP also display similar positivity to that of the controls for correct “remember” responses from around 200 ms until the end of the epoch in the RP region (Figure 6). This correct “remember” positivity, however, does not appear in the other scalp regions until around 300–400 ms after stimulus onset, but the “remember” old/new effect appears to be of similar magnitude for both participant groups.

Pairwise comparisons at each scalp location from the 300–500 ms time window revealed that the control group’s correct “remember” (*t*_(9)_ = 4.49, *SE* = 0.133, *p* = 0.001) responses were more positive than correct rejections at the LP region. Correct “remember” [*t*_(9)_ = 2.33, *SE* = 0.126, *p* = 0.042] responses were also more positive than correct rejections over the left anterior (LA) location. We found no correct “remember” old/new effects at any of the four a-priori scalp locations in the DP group between 300–500 ms.

In the 500–700 ms time window, pairwise comparisons revealed that correct “remember” responses at LP [*t*_(9)_ = 3.398, *SE* = 0.27, *p* = 0.008] and RP [*t*_(9)_ = 3.807, *SE* = 0.315, *p* = 0.004] regions were more positive than correct rejections in the control group. We also found that ERPs for correct “remember” responses were more positive than those for correct rejections [*t*_(9)_ = 2.487, *SE* = 0.345, *p* = 0.042] in the DP group over only the RP of the scalp.

This pattern of ERPs for control participants is consistent with previous research finding correct “remember” old/new effects over posterior and anterior scalp sites between 300–700 ms (Yovel and Paller, 2004; MacKenzie and Donaldson, 2007). The appearance of correct “remember” old/new effects, however, appear to be delayed in those with DP due to enhanced positivity appearing in the later time window only. This effect also seems quantitatively smaller in the DP group when compared to the controls as indicated by the positivity being restricted only to the RP of the scalp.

#### Recollection vs. familiarity

No significant differences were found between correct “remember” or correct “know” responses in the 300–500 ms time window for either of the two participant groups.

Further analyses on the controls between 500–700 ms revealed enhanced positivity for correct “remember” compared to correct “know” responses at RP [*t*_(9)_ = 4.667, *SE* = 0.298, *p* = 0.001] and right anterior (RA) [*t*_(9)_ = 2.483, *SE* = 0.347, *p* = 0.035] locations. We also found that ERPs for correct “remember” responses were more positive than “know” [*t*_(7)_ = 2.84, *SE* = 0.27, *p* = 0.025] responses in the DP group at the RP location.

This suggests that recollection is a much stronger signal in comparison to familiarity in the control group, but only over the right hemisphere. ERP differences in the DP group were again restricted to the posterior of the scalp, with this enhanced positivity for recollection to familiarity appearing only over the right parietal region of the scalp.

It is possible that differences between the two groups with regard to significant old/new effects were only due to differential power to detect these effects (due to different sample sizes and trial numbers). To rule this possibility out, we repeated the analyses after removing the two control participants with the fewest correct “know” responses and then matched the average trial numbers between the two groups. These analyses revealed the same pattern of results.

#### ERP effects commonly associated with familiarity for words and objects

Visual inspection of Figure 3 suggests the appearance of a frontal correct “know” old/new effect over the furthermost mid and left frontal sites in those with DP. Intriguingly, this frontal effect does not appear in the controls. A “know” old/new effect over frontal sites between 300–500 ms has previously been associated with familiarity of objects, pictures and words (e.g., Curran, 2000; Maratos et al., 2000; Tsivilis et al., 2001; Duarte et al., 2004; Groh-Bordin et al., 2006), but not generally for faces (Yovel and Paller, 2004; MacKenzie and Donaldson, 2007; Herzmann et al., 2011). Knowing that previous research (Curran, 2000; Maratos et al., 2000; Tsivilis et al., 2001; Duarte et al., 2004; Groh-Bordin et al., 2006) has identified this effect as occurring between the frontal and polarfrontal regions of the scalp, and visually inspecting where this effect was apparent in our data, we averaged the electrodes (C17, C18, C19, C27 and C28) to form a post-hoc region of interest: inferior mid left anterior (ILA). We also created an additional two regions of interest to more robustly confirm any possible effects using the exact frontal electrodes (Left Frontal (LF): C27, C29 and C32; Right Frontal (RF): C16, C14 and C10) as used by previous research (Duarte et al., 2004). The Duarte et al. (2004) study was chosen as the authors used visual objects which appeared to most closely match the stimuli used in the present study.

**Figure 7:**
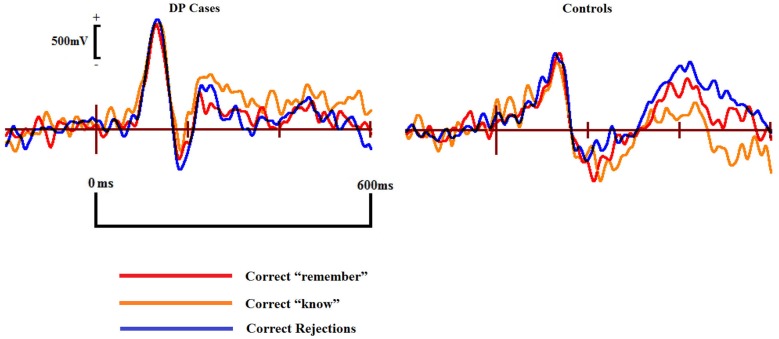
**ERPs of correct “remember” and “know” responses compared to correct rejections in the DP cases (left) and controls (right) from the inferior mid left anterior (ILA) region associated with familiarity for objects and words**.

Figure 7 displays the ERPs from the ILA region where we identified the apparent frontal positivity related to correct “know” responses. The three waveforms for correct responses appear qualitatively similar within the individuals with DP, suggesting a similar underlying cognitive process being engaged when making recognition judgments of a face in DP. Qualitative differences are clearly apparent when these waveforms from the DP group are compared to the correct response waveforms from the control group. Differences such as these suggest that the two groups are possibly engaging in different cognitive processes when making face recognition judgments.

To better assess the apparent frontal old/new effect for individuals with DP, and in an effort to look for any possible familiarity effects normally associated with objects, pictures and words, we conducted mixed within-between subject ANOVAs on the ILA region with factors of Correct Response (“remember”, “know”, “new”) and Group (control, DP) across the 300–500 and 500–700 ms time windows.

In the 300–500 ms time window over the ILA region, we found a significant interaction for Response × Group [*F*_(2,32)_ = 0.126, *MSE* = 0.0846, *p* = 0.022]. Pairwise comparisons revealed that correct “know” responses at the ILA site [*t*_(7)_ = 2.88, *SE* = 0.14, *p* = 0.024] were significantly more positive than correct rejections in the DP group. Conversely, correct rejections were significantly more positive than the correct “know” responses at this site in the control group [*t*_(9)_ = 2.88, *SE* = 0.073, *p* = 0.024]. Independent samples *t*-tests revealed that the magnitude of the correct “know” effect was greater in the DP group than in the controls [*t*_(16)_ = 3.24, *SE* = 0.26, *p* = 0.005]. Repeating these analyses using the electrodes examined by previous research (Duarte et al., 2004) confirmed these effects at the LF, but not RF, region.

In the 500–700 ms time period we found a significant interaction for Response × Group [*F*_(2,32)_ = 0.025, *MSE* = 0.012, *p* = 0.033]. Paired samples *t*-tests revealed a positive correct “know” old/new effect [*t*_(7)_ = 2.91, *SE* = 0.29, *p* = 0.022] in the DP group. No differences were found between any of the control waveforms in this time window. Between group comparisons revealed that this “know” old/new effect was larger in the DP group [*t*_(16)_ = 2.67, *SE* = 0.46, *p* = 0.017]. As with the earlier time window, these effects were confirmed at the LF, but not the RF, location.

We repeated the analyses after removing the two control participants with the fewest correct “know” responses and matching the average trial numbers between the two groups. We ranked the DP participants by the number of their correct “know” responses and separately ranked the controls in the same manner. We then matched each DP participant with their respectively ranked control participant, and reduced the number of trials for each DP participant to that of their matched control participant. The selection of which trials to remove was decided at random by a Python script. This was possible for 7 of the DP cases; one control participant had more correct “know” responses than their matched DP case, in this instance, the control participant had their trial numbers reduced to match the DP participant. These analyses revealed the same pattern of results.

These results suggest that when making recognition judgments, the DP group process faces using a neural pathway commonly associated with words, objects and pictures (e.g., Curran, 2000; Maratos et al., 2000; Tsivilis et al., 2001; Duarte et al., 2004; Groh-Bordin et al., 2006). Familiarity in DP thus appears to be driven by this object related recognition pathway. The controls, however, exhibit no evidence that they process faces using this route, instead it appears that they use routes commonly associated with intact face recognition abilities (Yovel and Paller, 2004; MacKenzie and Donaldson, 2007).

## Discussion

We examined recognition memory for previously unknown faces in both control participants and individuals with DP. Previous research has identified face recognition impairments in individuals with DP (e.g., Duchaine and Nakayama, 2006), but we are not aware of any previous attempts to assess the roles of familiarity and recollection for recognition performance in this group. We used an R/K recognition memory paradigm to measure the relative contributions of recollection, inferred from “remember” responses, and familiarity, inferred from “know” responses, to recognition memory for faces. We also obtained EEG recordings to identify the neural mechanisms involved in recognizing recently encountered faces in these two groups. We found that individuals with DP exhibited a variety of behavioral deficits in recognition memory and also differed in their electrophysiological response to test stimuli from individuals with normal face processing abilities. Specifically, in individuals with DP we observed (a) a relatively low proportion of “remember” responses and corresponding high proportion of “know” responses (suggestive of low levels of recollection); (b) a relatively high proportion of false alarms; (c) an apparent lack of a posterior familiarity ERP old/new effect commonly associated with faces as evidenced by similar waveforms for correct “know” responses and correct rejections across all time windows; (d) the appearance of a frontal “know” ERP old/new effect commonly associated with familiarity for objects, pictures and words (but not faces); and (e) a delay in the appearance of a recollection related ERP old/new effect as evidenced by ERPs for trials with correct “remember” responses only appearing more positive than those for trials with correct rejections in the later time window.

### Behavioral findings

In agreement with previous research we found that individuals with DP have a general impairment for recognizing faces when compared to controls. This impairment was driven by a decreased ability to correctly identify a previously seen face, but also through difficulties in correctly identifying a previously unknown face as new; these problems drive the DP group’s diminished capacity to discriminate old from new faces in comparison to the controls. For control participants this task was very easy as evidenced by their extremely high discriminability score, whereas individuals with DP exhibited considerable difficulties, especially for “know” responses which did not discriminate at all between old and new items. Controls identified faces on the basis of recollection in the vast majority of trials, utilizing familiarity much less frequently, as indicated by their relatively high proportion of “remember” rather than “know” responses. Conversely, among individuals with DP the proportions of correct “remember” and “know” responses were about equal. Even though individuals with DP exhibited a much higher false alarm rate than controls, both groups made predominantly “know” responses in this category, suggesting that the similar proportion of correct “remember” and “know” responses in individuals with DP might reflect a specific impairment in recollection rather than a general inability to distinguish between “remember” and “know” responses.

Dual process models of recognition memory purport that familiarity is a faster process than recollection (Yonelinas, 2002), and as such one would expect “know” responses to be faster than “remember” responses—a pattern opposite to that we observed. This discrepancy, however, can be explained by the quality of the distinct phenomenological experiences of recollection and familiarity. It is entirely possible that “remember” and “know” response times do not accurately reflect the actual temporal activation of recollection and familiarity, but rather the speed with which a participant can be confident enough to make a decision (Dewhurst and Conway, 1994; Dewhurst et al., 2006). For example, a participant might respond “remember” the instant a contextual detail is recollected due to the strength of evidence associated with this information. On the other hand, a feeling of familiarity without context may require extra time to elicit a “know” response. Under these circumstances, the dual process model’s assumption that familiarity is activated earlier than recollection is still compatible with the behavioral results of faster remember response times observed with the R/K procedure. It should be noted that it is remarkable that any RT differences exist at all; participants could only make a recognition response during a prompt screen which appeared after the face had already been displayed onscreen for 2000 ms.

What reasons could there be for the above differences between those with DP and normal face processing abilities? One explanation might be that of facial distinctiveness, or at least perceived facial distinctiveness, affecting recognition. Previous research has suggested that remember responses are primarily influenced by the distinctiveness of a face, with increasing distinctiveness leading to more recollected experiences (Dewhurst et al., 2005). Increasing distinctiveness has also been indicated as causing fewer false alarms (Light et al., 1979). It has been shown that some individuals with DP display random patterns when rating distinctiveness (Carbon et al., 2010), thus DP cases might have an inability to pick up on the subtle cues from a face that aid recollection. While those with DP possibly appear incapable of deciding distinctiveness in a similar fashion to controls, it would be interesting to see if distinctiveness, at least with regard to how those with DP perceive it, could influence later recognition performance. For example, is subsequent recognition performance for faces rated as distinctive at study by individuals with DP more accurate compared to faces rated as not distinctive, and if so, is this generally through the use of recollection? If recollection is primarily aided by distinctiveness, and that those with DP are incapable of making reliable distinctiveness judgments, then it does raise the question on what “remember” responses in individuals with DP are based. It might be interesting to see if other factors identified in face recognition are being used by those with DP, such as attractiveness, memorability, typicality or how much each face reminds them of someone they already know (Dewhurst et al., 2005).

Increasing usage of familiarity in discrimination tasks has been linked with face typicality, that is, how much a face looks like an average face (Vokey and Read, 1992; Dewhurst et al., 2005). Typicality and distinctiveness have been proposed to be opposite ends of a continuum upon which faces can be found (Johnston et al., 1997). Valentine (1991) formalized this idea into a face-space model, a multidimensional space whereby faces are located dependent upon their characteristics, at the center of which is an average, or typical, exemplar face. Faces that appear to be more typical, or lacking in distinctive features, are grouped around the center of this space, whereby the increased density and similarity of the faces in this area makes it much more difficult to discriminate between them. These faces are suggested to increase familiarity recognition judgments for studied and unstudied faces due to familiarity. Faces found further away from this center, those that are more distinctive, are much less susceptible to false alarms and are increasingly identified by recollection (Dewhurst et al., 2005).

The DP group’s low discriminability scores and increased usage of familiarity suggests that face-spaces for individuals with DP are smaller than those in individuals with normal face processing abilities, effectively leading to faces being closer to the center. This would suggest some testable predictions: because the space within which individuals with DP place faces is diminished when compared to controls, those with DP should therefore be less susceptible to the face-space effects found in recollection and familiarity when faces are either morphed to appear more average or distinctive. For example, in those with intact face recognition abilities we should find large increases in recollection if we caricatured faces to make them appear more distinctive and fewer false alarms to such faces. In theory, the magnitude of these effects should be diminished, or possibly non-existent, in DP. Similarly, it should be possible to induce DP-like recognition memory behavior in those with intact face processing skills if we averaged faces to make them appear more typical. It would be interesting to see if doing so would then cause the electrophysiological signatures of recollection and familiarity in those with intact face recognition abilities to appear more similar to those observed in individuals with DP. Two studies have found some normal face-space effects in DP (Nishimura et al., 2010; Susilo et al., 2010) however the lack of a recognition memory paradigm measuring the contributions of recollection and familiarity in either experiment would suggest the need for further research.

### Electrophysiological findings

The electrophysiological results for the control participants replicate previous research (Yovel and Paller, 2004; MacKenzie and Donaldson, 2007) in finding anterior and posterior old/new effects for “remember” responses and only posterior effects for “know” responses. Taking “remember” responses as an index of recollection, and “know” responses as an index of familiarity, recollection ERP old/new effects in the controls appeared generally to occur over anterior and posterior sites in the 300–500 ms and over posterior sites in the 500–700 ms time windows. Familiarity ERP old/new effects appeared only over LP sites in both time windows. We also found ERPs for “remember” responses to be more positive over right hemisphere regions than those for “know” responses (which were indistinguishable from those for correct rejections). In the controls, the complete lack of an anterior familiarity effect similar to that typically found for objects and words (e.g., Curran, 2000; Maratos et al., 2000; Tsivilis et al., 2001; Duarte et al., 2004; Groh-Bordin et al., 2006) suggests that such effects in other studies using face stimuli (e.g., Curran and Hancock, 2007) might be driven by features that are not central to faces such as hair, clothing, jewelry, and other objects also present in the stimuli. Furthermore, we also found agreement with previous research that recollection related activity for faces was greater than that of the activity associated with familiarity (Yovel and Paller, 2004; MacKenzie and Donaldson, 2007), at least with regard to the right hemisphere.

The lack of recollection effects in the 300–500 ms time window for participants with DP could be due to a general delay in the neural processing of face stimuli relative to the control group. Parietal recollection old/new effects for objects and words generally do not become apparent until 500 ms after stimulus onset (e.g., Maratos et al., 2000; Tsivilis et al., 2001), so if individuals with DP processed faces like other objects, we would not expect a recollection effect earlier than 500 ms after stimulus onset. ERPs for correct “remember” responses, however, look qualitatively similar between the two groups which would be inconsistent with the delayed neural processing of face stimuli in our DP group. Alternatively, it might be the case that the recollection old/new effect in the 300–500 ms time window for the control group is distinct from the corresponding effect in the later time window. Whereas the early and late effect have commonly been assumed to both index recollection (Yovel and Paller, 2004), it has been suggested that the early parietal effect might instead index familiarity (MacKenzie and Donaldson, 2007). It seems plausible that feelings of familiarity precede or at least coincide with recollection, and the early correct “remember” ERP positivity we observed over the left hemisphere of control participants may thus reflect that of a familiarity signal. The absence of this ERP positivity in individuals with DP could be related to their lack of posterior ERP old/new effect for “know” responses: both could index a lack of familiarity for previously studied faces. Consistent with this explanation is the fact that early correct “remember” and “know” ERP waveforms are virtually identical over the LP region for control participants.

Looking at the later time window, we see a clear recollection ERP old/new effect in the DP cases, one that is similar topographically and in magnitude to that of the controls, at least over the RP region. This suggests that the phenomenological experience of recollecting a face in individuals with DP is intact despite the drastically reduced proportion of “remember” responses in this group. Dual process theories that view recollection as an all-or-nothing process (e.g., Yonelinas, 1994) would predict similar effect sizes for effects due to recollection. Despite the recollection old/new effect between the two groups appearing to be of similar magnitude over the right parietal region, the fact that no old/new effects were found over other scalp locations might suggest a quantitatively weaker recollection signal in those with DP. This lends tentative support to the proposal that recollection could be a graded, as opposed to discrete, process as suggested by some theories of recognition memory (Wixted, 2007; Wixted and Mickes, 2010).

In the present paradigm, we relied upon participants’ own self-generated details for recollection. There might be a concern that this method does not index recollection commonly experienced in the real world, such as that for names, occupations or places. The Yovel and Paller (2004) study found recollection ERP old/new effects related to self-generated details surrounding a face to be qualitatively similar to that of occupations; these recollection old/new effects were also topographically similar to those found here. This would suggest that recollection of self-generated information attached to a face is the same as semantic information provided from external sources. MacKenzie and Donaldson (2007), however, found a larger old/new effect when names were recollected in comparison to self-generated details. It would therefore be of interest to see whether the recollection deficits observed here in our DP group would continue to be observed when an objective measure of recollection, such as a name, is employed; if names were no different from other semantic information, then we should observe similar behavioral and electrophysiological abnormalities in DP to those observed for recollection here.

The face related posterior familiarity old/new effect, however, appears to be absent in the DP group, suggesting that the subjective experience leading to “know” responses might differ between the two groups. While those with intact face recognition clearly exhibit a posterior ERP old/new effect when experiencing familiarity for faces, those with DP appear to engage a familiarity route more commonly associated with object, picture and word recognition towards the front of the scalp. This is to our knowledge the first clear evidence that individuals with DP are not processing faces using a specialized, face-specific pathway, but are instead using a route more commonly associated with general objects. Even more interesting is that this pathway appears to be engaged by the DP group during all recognition judgments, as evidenced by the qualitative similarities between the three different correct response ERP waveforms at the frontal region. The ERP waveforms exhibited by the controls in all response categories were qualitatively different in comparison to the DP group, so much so that the correct “know” old/new effect was actually more negative in amplitude in the control group. This finding was a reversal of the correct “know” old/new effect found in the DP group at the same site. It thus appears that an attempt is made to engage the object familiarity process in parallel with the face related recollection experience in DP. These results offer an exciting insight as to why those with DP might be experiencing problems when trying to recognize a face; a face is not treated entirely as special, but also processed using a generic, object related pathway in the brain.

Some authors (e.g., Yovel and Paller, 2004) have suggested that the parietal familiarity and recollection old/new effects are reliant on similar neural generators, thus implying that recollection and familiarity are merely quantitatively different strengths of the same signal. Furthermore, it has also been suggested that the frontal familiarity old/new effect merely reflects conceptual priming (Yovel and Paller, 2004; Paller et al., 2007; Voss et al., 2012) due to the existence of a base level of meaning for stimuli such as words (Maratos et al., 2000) and everyday objects (e.g., Duarte et al., 2004) in recognition memory experiments. The dissociation between the parietal familiarity and recollection effects in those with DP, and the appearance of the frontal familiarity effect commonly associated with objects and words, lends support to the proposal that the posterior familiarity and recollection old/new effects for faces are being driven by dissociable processes. Further to this, that previously novel faces, stimuli highlighted as not susceptible to the conceptual priming problem (Yovel and Paller, 2004), should elicit a frontal familiarity effect in the DP group suggests that the conceptual priming hypothesis is incorrect. Instead, our results would appear to add support to the notion that the mid-frontal ERP effect does actually index a generic familiarity process.

An alternative view, however, might be able to reconcile our data with the conceptual priming hypothesis. Voss and Paller (2007) found that the magnitude of the mid-frontal old/new effect increased in response to increasing ratings of meaningfulness for shapeless blobs; this supports the view that the mid-frontal old/new effect is merely an index of conceptual priming. If our DP cases are not entirely processing faces as faces through typical routes, as evidenced by the lack of a parietal familiarity old/new effect, then it might be the case that they are attempting to find some form of meaning in the faces instead. By trying to find some meaningful way to examine the faces, rather than treating them merely as faces, our DP participants might therefore be rating faces as familiar on the basis of conceptual priming. Our data would therefore still be compatible with the conceptual priming hypothesis if this were found to be the case. Regardless of the underlying neural cause of the mid-frontal old/new effect, it would appear that this pathway is driving familiarity based recognition judgments in our DP group.

It should be noted that although not significant, the topographical figures and waveforms do appear to hint at a possible familiarity effect in the DP group that is qualitatively similar to the controls, albeit hugely dissipated. The lack of differences between correct “know” and correct rejection waveforms at posterior sites might be an index of the difficulty that those with DP are finding at discriminating between the old and new faces; the face related familiarity signal elicited by a face may be so weak for individuals with DP in comparison to the controls that it is incapable of creating a large enough effect in the waveforms to be statistically apparent here. Maybe due to this weakness in the face-specific familiarity route, those with DP then engage the more general object and word familiarity route to aid recognition.

No previous recognition memory study for faces has found a modulation of this parietal familiarity effect. This occurrence in the present study suggests that familiarity for faces could be modulated in a similar way to the anterior familiarity effect seen for objects and words. Future research could employ experimental manipulations to uncover whether these familiarity effects can be modulated through increasing levels of familiarity or confidence. Another possibility could be that familiarity for faces is linked to the same underlying process that detects distinctiveness in faces; the fact that those with DP might be incapable of making distinctiveness judgments in a similar fashion to those with intact face processing abilities (Carbon et al., 2010) could be due to the fact that they are utilizing an object/word route to make such judgments. The nature of the parietal face related familiarity effect has been largely ignored by recognition memory researchers and is an area ripe for study, not only in DP, but also in those with intact face processing abilities. Combined with experimental manipulations of facial distinctiveness, they could provide investigators with a powerful framework within which to elucidate the possible causes of recognition deficits in DP.

## Conclusions

The present study examined recognition memory for previously unknown faces in DP using an R/K paradigm. From our findings it is clear that there are a range of abnormalities in recognition memory for faces in individuals with DP. These findings supply compelling evidence that future DP researchers should take the relative contributions of recollection and familiarity into consideration when designing studies investigating face recognition. Our electrophysiological results give the first clear evidence that individuals with DP process faces like other objects and we propose that the associated impairments in performance may be related to difficulties in judging distinctiveness and/or typicality of previously unknown faces (Carbon et al., 2010). This finding would not have been apparent from the behavioral results alone and highlights the importance of combining different approaches when investigating face recognition deficits in DP.

The present research also has important implications when diagnosing, and testing treatments of, DP. Further work is required to discover the extent to which those with DP and normal face recognition abilities are utilizing familiarity and recollection when completing the widely used CFMT; a primary tool for diagnosing DP. Around half of all individuals that contact us reporting problems with faces fail to meet the criteria for a diagnosis of prosopagnosia when using the CFMT. The CFMT simply asks participants to pick out a target face from a choice of three faces, with no measure as to how this decision was made. The basis on which those with intact face recognition abilities are identifying faces on the CFMT is as yet unknown, although one could imagine it is primarily through the use of recollection. Those that meet the criteria for a diagnosis on the CFMT might be more reliant on, as our study has demonstrated, a weakened recollection signal and abnormal familiarity route. It is possible that the individuals that report problems with faces, yet fail to meet a diagnosis, might be in some as yet undetected group exhibiting quantifiably distinct recognition processes. If we were to incorporate the R/K or confidence response options into the CFMT, we might find differences between those who report problems yet score within the normal range on the CFMT and others who report no such difficulties. Our findings provide new insights into recognition memory for faces in DP and should guide future research and attempts to improve diagnosis.

## Conflict of interest statement

The authors declare that the research was conducted in the absence of any commercial or financial relationships that could be construed as a potential conflict of interest.
